# Clonal evolution in liver cancer at single-cell and single-variant resolution

**DOI:** 10.1186/s13045-021-01036-y

**Published:** 2021-02-02

**Authors:** Xianbin Su, Linan Zhao, Yi Shi, Rui Zhang, Qi Long, Shihao Bai, Qing Luo, Yingxin Lin, Xin Zou, Shila Ghazanfar, Kun Tao, Guoliang Yang, Lan Wang, Kun-Yan He, Xiaofang Cui, Jian He, Jiao-Xiang Wu, Bo Han, Bin Yan, Biao Deng, Na Wang, Xiaolin Li, Pengyi Yang, Shangwei Hou, Jielin Sun, Jean Y. H. Yang, Jinhong Chen, Ze-Guang Han

**Affiliations:** 1 Key Laboratory of Systems Biomedicine (Ministry of Education), Shanghai Center for Systems Biomedicine, Shanghai Jiao Tong University, Shanghai, China; 2 Bio-X Institutes, Key Laboratory for the Genetics of Developmental and Neuropsychiatric Disorders, Shanghai Jiao Tong University, Shanghai, China; 3 Department of General Surgery, Huashan Hospital and Cancer Metastasis Institute, Fudan University, Shanghai, China; 4 Key Laboratory for Regenerative Medicine (Ministry of Education), School of Biomedical Sciences, Faculty of Medicine, The Chinese University of Hong Kong, Hong Kong, Hong Kong SAR China; 5 School of Mathematics and Statistics and Charles Perkins Center, The University of Sydney, Sydney, Australia; 6 Cancer Research UK Cambridge Institute, Li Ka Shing Centre, University of Cambridge, Robinson Way, Cambridge, CB2 0RE UK; 7 Department of Pathology, Tongren Hospital, Shanghai Jiao Tong University School of Medicine, Shanghai, China; 8 Department of Urology, Renji Hospital, Shanghai Jiao Tong University School of Medicine, Shanghai, China; 9 Hongqiao International Institute of Medicine, Tongren Hospital, Shanghai Jiao Tong University School of Medicine, Shanghai, China; 10 Department of General Surgery, Shanghai General Hospital, Shanghai Jiao Tong University School of Medicine, Shanghai, China; 11 Computational Systems Biology Group, Children’s Medical Research Institute, The University of Sydney, Westmead, NSW 2145 Australia

**Keywords:** Hepatocellular carcinoma, Genetic heterogeneity, Somatic mutation, Clonal structure, Tumor evolution

## Abstract

Genetic heterogeneity of tumor is closely related to its clonal evolution, phenotypic diversity and treatment resistance, and such heterogeneity has only been characterized at single-cell sub-chromosomal scale in liver cancer. Here we reconstructed the single-variant resolution clonal evolution in human liver cancer based on single-cell mutational profiles. The results indicated that key genetic events occurred early during tumorigenesis, and an early metastasis followed by independent evolution was observed in primary liver tumor and intrahepatic metastatic portal vein tumor thrombus. By parallel single-cell RNA-Seq, the transcriptomic phenotype of HCC was found to be related with genetic heterogeneity. For the first time we reconstructed the single-cell and single-variant clonal evolution in human liver cancer, and dissection of both genetic and phenotypic heterogeneity will facilitate better understanding of their relationship.

## To the Editor,

**Fig. 1:**
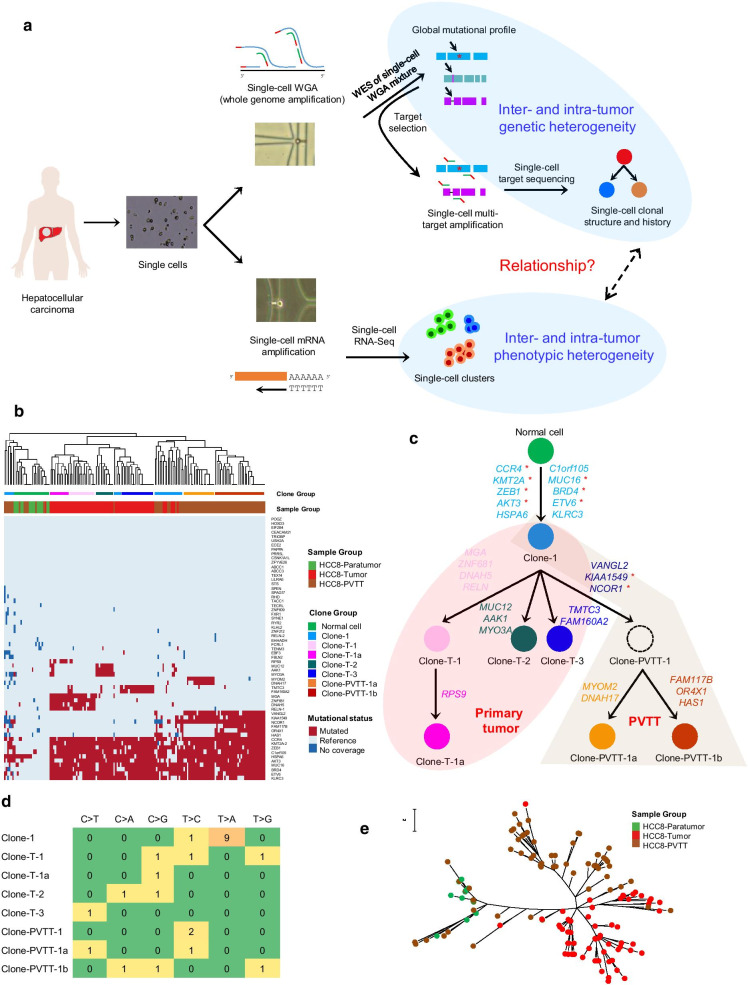
Single-cell analysis revealed a common origin but independent evolution of primary and metastatic liver tumors. **a** Overview of the single-cell analysis strategy of human liver cancer. **b** Mutational status of SNV/INDEL sites in single cells from paratumor, primary tumor and PVTT tissues in HCC8. **c** Clonal evolution in HCC8 with genes mutated at each step shown. Dashed circle: virtual ancestor clone in PVTT. *COSMIC Cancer Gene Census catalogued driver genes. **d** Statistics of nucleotide substitution types for clone-specific point mutations newly acquired from the most recent ancestor in HCC8 as shown in (**c**). **e** Maximum parsimony tree of single cells from HCC8 based on nucleotide sequences at the target sites. Scale bar: nucleotide substitution rate

The genetic heterogeneity in hepatocellular carcinoma (HCC) has been extensively studied by bulk or multi-region sequencing [1, 2], and more recently at single-cell sub-chromosomal scale [3, 4]. Analysis at single-variant resolution, however, is still lacking. To address this issue, here we employed a single-cell strategy to dissect the single-variant clonal structure of HCC, and investigate the relationship between genetic and phenotypic heterogeneity (Fig. 1a). A total of 5 HCC patients (HCC1, HCC2, HCC5, HCC8 and HCC9) were analyzed, including one (HCC8) with both primary tumor and the intrahepatic portal vein tumor thrombus (PVTT) (Additional file 1: Supplementary Methods, and Additional file 2: Fig. S1).

Great inter-tumor genetic heterogeneity of HCC was revealed by pseudo-bulk whole exome sequencing (WES), with different somatic mutations and mutational signatures observed among patients (Additional file 2: Fig. S2 and Additional file 3: Table S1). To further explore the intra-tumor heterogeneity, ~ 60 mutation sites (Additional file 4: Table S2) were then selected from each patient for target sequencing on single cells. High quality single-cell mutation data were obtained with good correlations between mutated cell fractions and WES-derived variant allele frequency values, as well as with low allele drop-out rates in most samples (Additional file 2: Fig. S3).

The clonal structures of liver tumor tissues were then uncovered by single-cell mutational profiles. Both HCC1 and HCC2 exhibited a single-clone structure with limited heterogeneity, while a multi-clone structure was observed in HCC9 (Additional file 2: Fig. S4). The evolutionary history of tumor cells in HCC9 was reconstructed by employing mutation combination analysis in single cells. The initiated cell was malignantly transformed to the founder Clone-1 as 9 genes were mutated, where 4 of them are COSMIC Census drivers [5]. Other clones were then derived from Clone-1 by acquisition of extra subclonal mutations.

A common origin but independent evolution pattern was observed in primary tumor and metastatic PVTT. Single cells from both tumor tissues in HCC8 shared mutations on 10 genes, where 7 of them are Census drivers: *CCR4*, *KMT2A*, *ZEB1*, *AKT3*, *MUC16*, *BRD4* and *ETV6* (Fig. 1b, c). Clone-1 with the 10 shared mutations represented a common origin, and other cells in both tumor tissues had divergent extra mutations (Fig. 1c). This implied an early stage metastasis followed by independent evolution, consistent with recent observation of early metastatic seeding in other types of solid tumor [6]. Two clones within PVTT shared PVTT-private clonal mutations on 3 genes, where *KIAA1549* and *NCOR1* are Census drivers related to tumorigenesis [7, 8]. Mutations on these two genes thus might be metastasis-related early genetic events. Interestingly, nine out of ten mutations in Clone-1 were T > A substitutions (Fig. 1d) related to carcinogen aristolochic acids [9], consistent with previous suggestion of early rather than late exposure for HCC development [10]. This suggested that early genetic event during liver tumorigenesis may be related to specific etiology. The phylogenetic tree of single cells also supported the multi-clone structure in HCC8 (Fig. 1e), which represented genuine tumor phylogeny different from that derived from bulk or multi-region sequencing [11].

**Fig. 2:**
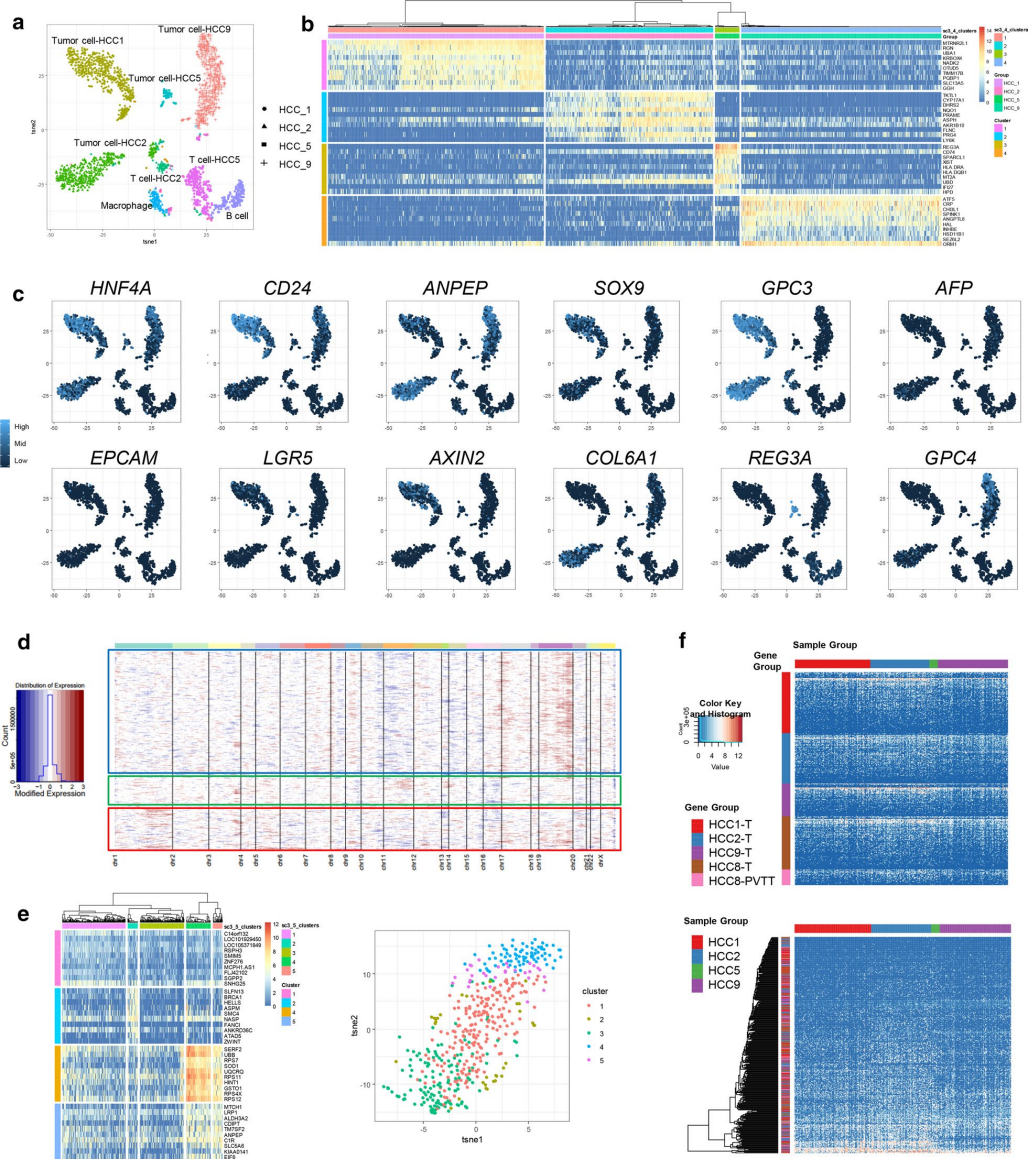
Genetic and phenotypic heterogeneity are showing consistence in liver cancer. **a** Cell clusters in scRNA-Seq analysis. **b** Heatmap showing patient-specific tumor marker genes. **c** The expression patterns of representative markers. **d** Copy number changes inferred from global transcriptomic profile of single cells in HCC9, with boxes highlighting sub-populations. **e** Heatmap (left) and t-SNE plot (right) showing the sub-populations in HCC9 based on differentially expressed genes. **f** The expression patterns of genes mutated in each HCC sample in scRNA-Seq data. Genes were grouped by tissue origin in the upper panel, and clustered by expression patterns in the lower panel

The inter- and intra-tumor genetic heterogeneity in HCC were found consistent with phenotypic heterogeneity by parallel single-cell RNA-Seq (Additional file 2: Fig. S5). Tumor cells from 4 patients formed separate clusters, illustrating patient-specific transcriptomic profiles (Fig. 2a–c and Additional file 2: Fig. S6a, b). For intra-tumor heterogeneity, the sub-chromosomal scale copy number inference from global transcriptomic profiles in HCC1 or HCC2 were quite similar within each patient, consistent with their single-clone structures (Additional file 2: Fig. S6c, d). For HCC9, both copy number inference and differentially expressed gene analysis identified tumor sub-populations, echoing its relatively higher genetic heterogeneity (Fig. 2d, e). Interestingly, the genes mutated specifically in each tumor showed similar expression patterns among single cells from different patients, and clustering of those genes exhibited a mixture of patient origin (Fig. 2f). This suggested that the tumor-specific mutations in HCC might cause phenotypic heterogeneity by altering the expressions of other genes rather than their own. The direct link between genetic and phenotypic heterogeneity in HCC, however, still await further clarification with new single-cell multi-omics tool that could co-detect point mutation and gene expression [12].

In summary, here we reconstructed single-cell clonal evolution in human liver cancer at single-variant resolution. The common origin but independent evolutionary fate for primary and metastatic liver tumors observed here may help understanding liver cancer progression, and single-cell dissection of both genetic and phenotypic heterogeneity will provide information for their functional linkage.

## Supplementary Information

**Additional file 1**. Supplementary Methods.

**Additional file 2**: **Figure S1**. Overview of the single-cell analysis strategy of human HCC. **Figure S2**. Single-cell mixture WES revealed inter-tumor genetic heterogeneity of HCC. **Figure S3**. High quality single-cell mutation data were obtained by target sequencing. **Figure S4**. Single-cell clonal structures of HCC1, HCC2 and HCC9 based on point mutations. **Figure S5**. scRNA-Seq revealed the constituent cell types of HCC. **Figure S6**. scRNA-Seq revealed the inter-tumor and intra-tumor heterogeneity of HCC. **Figure S7**. Schematic representation of major findings in this study.

**Additional file 3**: **Table S1**. Exonic mutations in single-cell WGA mixtures from HCC. This table shows all the exonic mutations derived from WES of single-cell WGA mixtures from HCC1-T, HCC2-T, HCC5-T, HCC9-T, HCC8-T and HCC8-PVTT. Mutations were called with GATK, and annotated with ANNOVAR and Oncotator. SNPs were filtered using dbSNP141 and 1,000 Genomes Project (v3) database. The synonymous mutations were further filtered. This table is related to Fig S2.

**Additional file 4**: **Table S2**. Mutation sites and amplification primers for single-cell target sequencing. This table shows the full list of target sites and PCR primers used in single-cell target amplification for HCC1, HCC2, HCC9 and HCC8. The primer sequences include the adaptor sequences that can be used for downstream library preparation with illumina Nextera XT Index Kit and multi-plexed sequencing.

## Data Availability

The sequencing data have been deposited in NCBI GEO and SRA database under accession numbers GSE146115 and PRJNA606993.

## Abbreviations

HCC: Hepatocellular carcinoma
PVTT: Portal vein tumor thrombus
WES: Whole exome sequencing
