# Supplementary material for: Clonal evolution in liver cancer at single-cell and single-variant resolution
Source: J Hematol Oncol. 2021 Feb 2;14:22. doi: 10.1186/s13045-021-01036-y (PMC7852352; doi:10.1186/s13045-021-01036-y)
Supplement: Supplementary file 1 — Additional file 1. Supplementary Methods. [file 13045_2021_1036_MOESM1_ESM.pdf]

Supplementary Methods

Clinical specimens

Both tumor and paratumor tissues were collected from a total of 5 HCC patients (HCC1, HCC2, HCC5, HCC8, and HCC9), including one (HCC8) with both primary tumor and the intrahepatic metastatic portal vein tumor thrombus (PVTT). The tissues were minced and digested in 0.05% collagenase IV (Sigma-Aldrich, C5138) at 37°C for 30 min with agitation. Centrifugations at 50× *g* for 2 min were repeated three times to enrich tumor cells, and a 40 μm-cell strainer (Corning, 352340) was used to remove undigested tissues. The single-cell suspensions were used for both single-cell whole genome amplification (WGA) and single-cell RNA-Seq (scRNA-Seq).

Single-cell WGA and exome sequencing

Single-cell WGA was done on 10~17 μm or 17~25 μm C1 DNA-Seq IFC (Fluidigm, 100-5763 or 100-5764) via multiple displacement amplification method using illustra GenomiPhi V2 DNA Amplification Kit (GE Healthcare, 25660031). The WGA products from single cells were mixed according to their tissue origin, and the exonic regions were captured with Agilent SureSelect Human All Exon v7 Kit (Agilent, 5191-4005). Whole exome sequencing (WES) was done on illumina HiSeq platform with 2 × 150 bp mode. Variant calling, functional annotation, SNPs filtering, and mutational signature analysis were done as previously described [1].

Selection of mutations for single-cell target sequencing

Putative clonal and subclonal mutations were selected from WES-derived mutation list, and the target sites were further narrowed down by checking mutation prevalence in cBioPortal collected HCC samples [2] and Gene Set Enrichment Analysis [3]. A total of 57, 56, 57 and 64 mutation sites were selected for HCC1, HCC2, HCC9 and HCC8, respectively. HCC5 was not used as many mutations had low variant allele frequency (VAF) values, suggesting low tumor cell purity which was confirmed by scRNA-Seq. Primers were designed to amplify these sites from single-cell WGA product, and the amplification specificities of the primers were confirmed to ensure one PCR product for each primer pair.

Single-cell target sequencing

A total of 480 cells were used for single-cell target sequencing, with 96, 96, 96 and 192 single cells from HCC1, HCC2, HCC9 and HCC8, respectively. WGA product from each single cell was used as PCR template for 5 parallel PCR reactions with ~10 primer pairs in each reaction. PCR cycling program was adopted from User Guide for Access Array System (Fluidigm, 100-3770) for even amplification of multiple targets. For each single cell, PCR products from the 5 reactions were mixed for library preparation using FastStart High Fidelity PCR System dNTPack (Roche, 3553400001) and Nextera XT Index Kit (illumina, FC-131-2001). Single-cell libraries were mixed for sequencing on illumina NextSeq500 platform with 1 × 151 bp mode, and de-multiplexed based on their index combinations. The sequencing reads were

mapped to GRCh37/hg19 with Tophat using default parameters, and numbers of the reads with reference or mutation site were counted.

Single-cell mutational status and clonal structure analysis

The following criteria were used to determine the mutational status of a given target site: if reads <3, it was defined as site with no coverage; if reads >=3, more than 1 read with mutation, and VAF >=0.1, then it was defined as mutated site; other conditions were defined as reference site. All target sites in single cells were further confirmed by manual inspection in Integrative Genomics Viewer [4]. We filtered genetic variations with poor coverage, and also removed those found in both paratumor and tumor cells which may be SNPs. Finally, 54/57, 55/56, 56/57 and 61/64 mutations were kept for HCC1, HCC2, HCC9 and HCC8, respectively. For cell filtering, we removed single cells with less than half of the sites covered, and 71/96, 60/96, 91/96, 177/192 cells were kept for HCC1, HCC2, HCC9 and HCC8, respectively.

We selected drop-out of normal allele for ADO assessment, which will generate VAF values approximating 1. After filtering sites with genuine homogenous mutations, except for HCC2 with rate of 56.9%, all samples showed good results with genetic variations having VAF values of 0.95~1 occupying 15.8%, 6.3%, 2.1% and 6.3% of total variations for HCC1, HCC9, HCC8-T and HCC8-PVTT.

The combination of mutations enabled inference of clones and clone-specific mutations in each patient, which could reconstruct their evolutionary relationship. For

HCC9, 17 cells were found to be mixture of more than one cell based on VAF analysis and removed before clonal analysis. Besides single-cell clonal analysis based on the mutational status, we also used nucleotide sequences at each site for evolutionary phylogenetic tree reconstruction. The combined sequences from all target sites in the single cells were aligned, and maximum parsimony tree was constructed using MEGA-X [5].

scRNA-Seq and data analysis

scRNA-Seq was done on 10~17 μm C1 mRNA-Seq HT IFC (Fluidigm, 101-4981). Single-cell libraries were pooled and sequenced on illumina HiSeq platform with 2 × 150 bp mode. The C1 mRNA Sequencing High Throughput Demultiplexer Script (Fluidigm) was used for de-multiplexing and generation of FASTQ files for each single cell. The sequencing reads were mapped to GRCh37/hg19 with Tophat using the default parameters, and the numbers of reads in each gene were counted. ERCC RNA Spike-in (Ambion, 4456740) was added as a technical control. SC3 was used for outlier identification and cell type identification [6]. Single-cell transcriptomes were filtered based on three parameters: total counts, total features, and ERCC count percentage. If all three parameters fall within 1 MAD (median of the absolute deviation) away from median then the cell was kept, and a total of 2064 cells from the 3200 cells passed the filtering. HCC8 samples were not analyzed because the size of tumor cell was out of the capture range of C1 HT IFC. Gene Ontology enrichment and transcriptional factor co-variance network analysis were done as previously described

[1], and copy number inference from single-cell global transcriptional profiles was done with inferCNV software (https://github.com/broadinstitute/inferCNV).

Supplementary references

1. Su X, Long Q, Bo J, Shi Y, Zhao LN, Lin Y, Luo Q, Ghazanfar S, Zhang C, Liu Q, et al: **Mutational and transcriptomic landscapes of a rare human prostate basal cell carcinoma.** *Prostate* 2020, **80:**508-517.

2. Gao J, Aksoy BA, Dogrusoz U, Dresdner G, Gross B, Sumer SO, Sun Y, Jacobsen A, Sinha R, Larsson E, et al: **Integrative analysis of complex cancer genomics and clinical profiles using the cBioPortal.** *Sci Signal* 2013, **6:**pl1.

3. Subramanian A, Tamayo P, Mootha VK, Mukherjee S, Ebert BL, Gillette MA, Paulovich A, Pomeroy SL, Golub TR, Lander ES, Mesirov JP: **Gene set enrichment analysis: a knowledge-based approach for interpreting genome-wide expression profiles.** *Proc Natl Acad Sci U S A* 2005, **102:**15545-15550.

4. Robinson JT, Thorvaldsdottir H, Winckler W, Guttman M, Lander ES, Getz G, Mesirov JP: **Integrative genomics viewer.** *Nat Biotechnol* 2011, **29:**24-26.

5. Kumar S, Stecher G, Li M, Knyaz C, Tamura K: **MEGA X: Molecular Evolutionary Genetics Analysis across Computing Platforms.** *Mol Biol Evol* 2018, **35:**1547-1549.

6. Kiselev VY, Kirschner K, Schaub MT, Andrews T, Yiu A, Chandra T, Natarajan KN, Reik W, Barahona M, Green AR, Hemberg M: **SC3: consensus clustering of single-cell RNA-seq data.** *Nat Methods* 2017, **14:**483-486.
