# Supplementary material for: Clonal evolution in liver cancer at single-cell and single-variant resolution
Source: J Hematol Oncol. 2021 Feb 2;14:22. doi: 10.1186/s13045-021-01036-y (PMC7852352; doi:10.1186/s13045-021-01036-y)
Supplement: Supplementary file 2 — Additional file 2: Figure S1. Overview of the single-cell analysis strategy of human HCC. Figure S2. Single-cell mixture WES revealed inter-tumor genetic heterogeneity of HCC. Figure S3. High quality single-cell mutation data were obtained by target sequencing. Figure S4. Single-cell clonal structures of HCC1, HCC2 and HCC9 based on point mutations. Figure S5. scRNA-Seq revealed the constituent cell types of HCC. Figure S6. scRNA-Seq revealed the inter-tumor and intra-tumor heterogeneity of HCC. Figure S7. Schematic representation of major findings in this study. [file 13045_2021_1036_MOESM2_ESM.pdf]

Supplementary Figures

Supplementary Fig. 1 Overview of the single-cell analysis strategy of human HCC.

Supplementary Fig. 2 Single-cell mixture WES revealed inter-tumor genetic heterogeneity of HCC.

Supplementary Fig. 3 High quality single-cell mutation data were obtained by target sequencing.

Supplementary Fig. 4 Single-cell clonal structures of HCC1, HCC2 and HCC9 based on point mutations.

Supplementary Fig. 5 scRNA-Seq revealed the constituent cell types of HCC.

Supplementary Fig. 6 scRNA-Seq revealed the inter-tumor and intra-tumor heterogeneity of HCC.

Supplementary Fig. 7 Schematic representation of major findings in this study.

a

Patient	Gender	Age	Stage	Tumor size	PVTT	Cell No.: WES	Cell No.: scTarget-seq	Cell No.: scRNA-Seq
HCC1	M	64	HCC, II~III	-	-	135	96	800
HCC2	M	66	HCC, II	10*6*5cm	-	123	96	800
HCC5	F	77	HCC, II	5*3*3cm	-	145	-	800
HCC8	M	64	HCC, II	4.5*4*3.5cm	√	257	192	-
HCC9	M	47	HCC, II	8.6*6*6cm	-	176	96	800

b

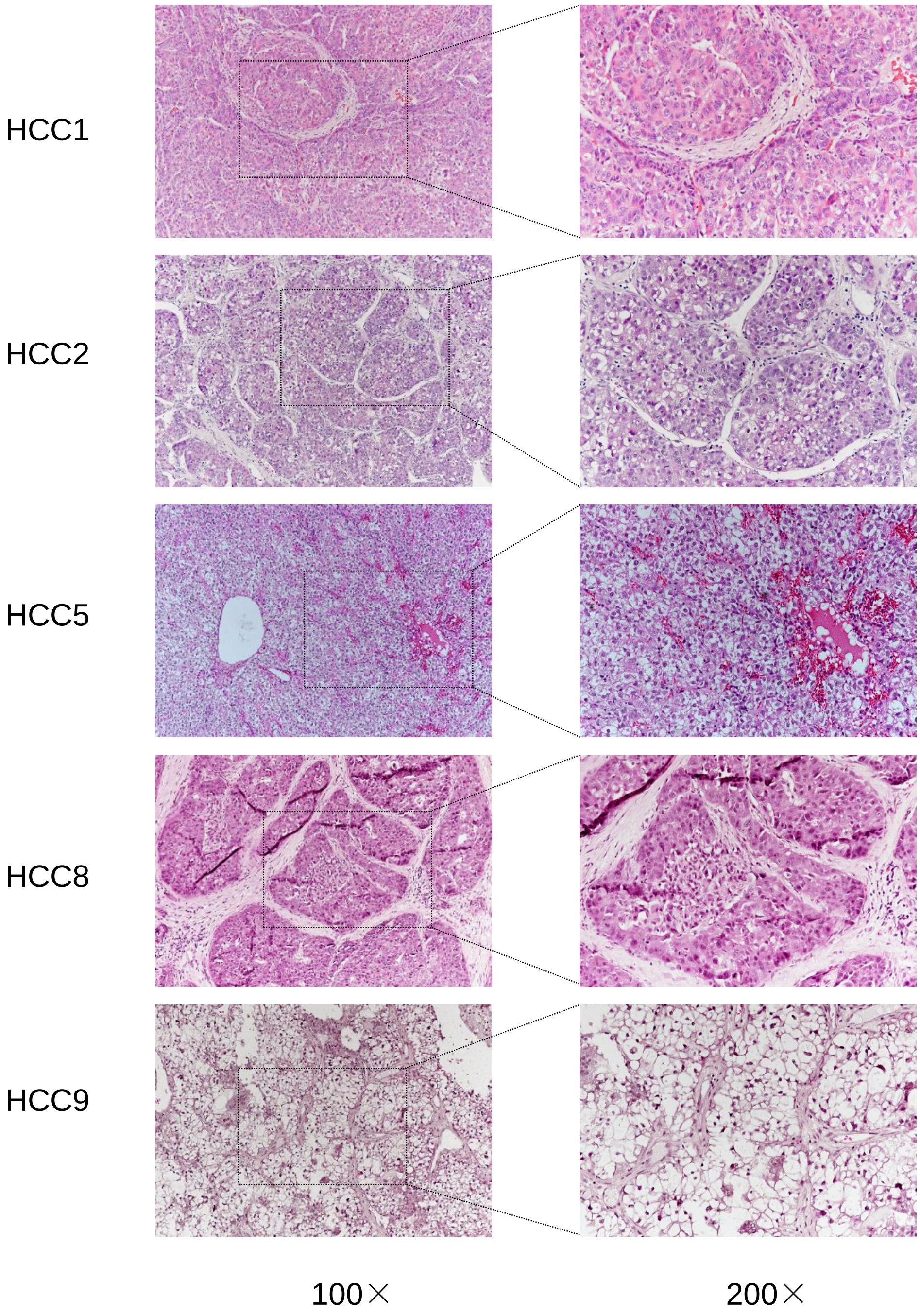

Supplementary Fig. 1 Overview of the single-cell analysis strategy of human HCC. **a** Patient information and investigation summary. **b** H&E staining of HCC tumor tissues.

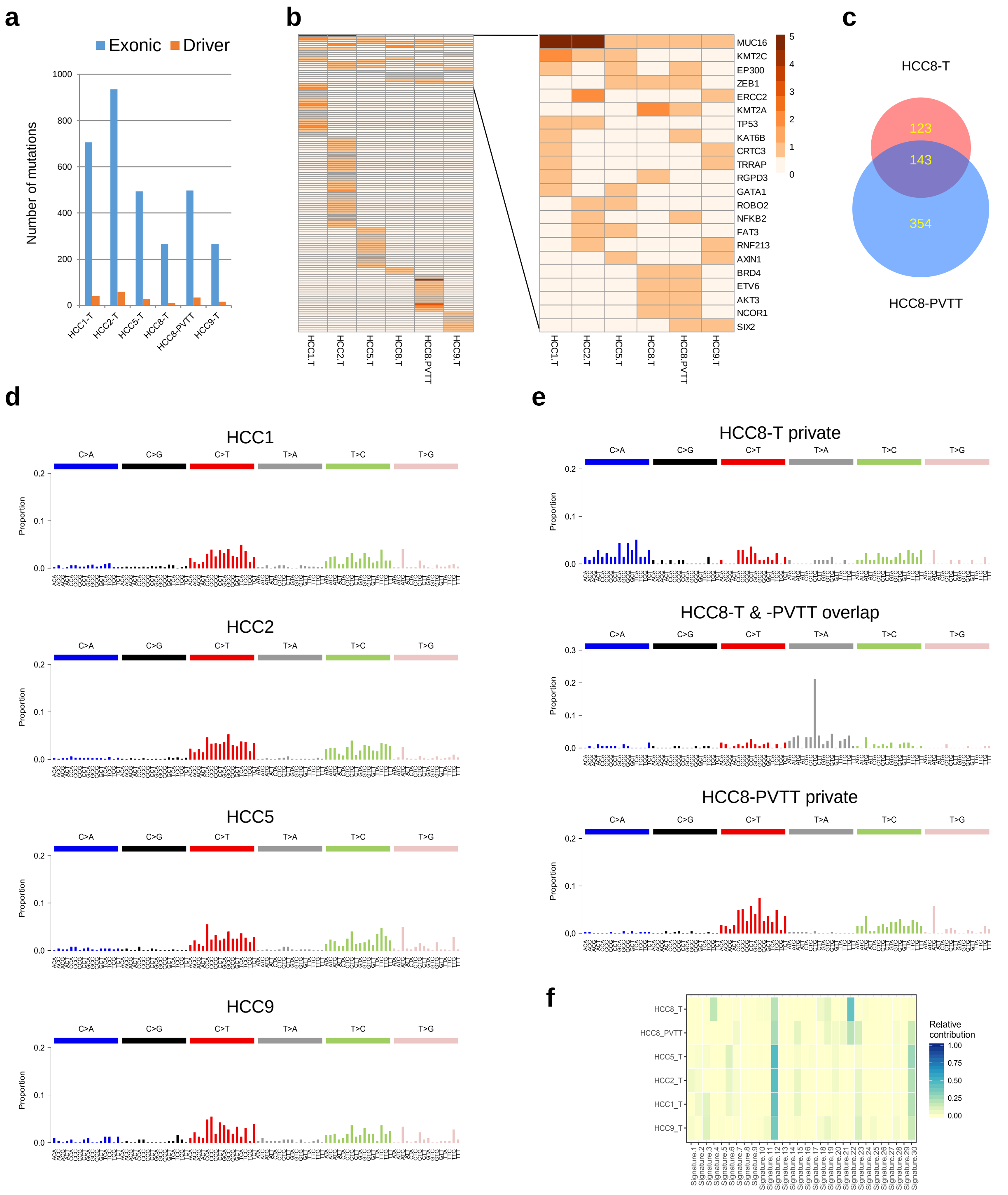

Supplementary Fig. 2 Single-cell mixture WES revealed inter-tumor genetic heterogeneity of HCC. **a** Number of exonic mutations and mutations on driver genes catalogued in the COSMIC Cancer Gene Census in HCC tumor tissues. **b** Heatmap showing the numbers of exonic mutations on driver genes by single-cell mixture WES. **c** Overlap of exonic mutations in primary tumor and PVTT of HCC8. **d** Mutational spectra of genetic variations in tumor tissues from HCC1, HCC2, HCC5 and HCC9. **e** Mutational spectra of genetic variations shared or privately detected in primary tumor and PVTT of HCC8. **f** Contributions of COSMIC signatures to the HCC mutational spectra.

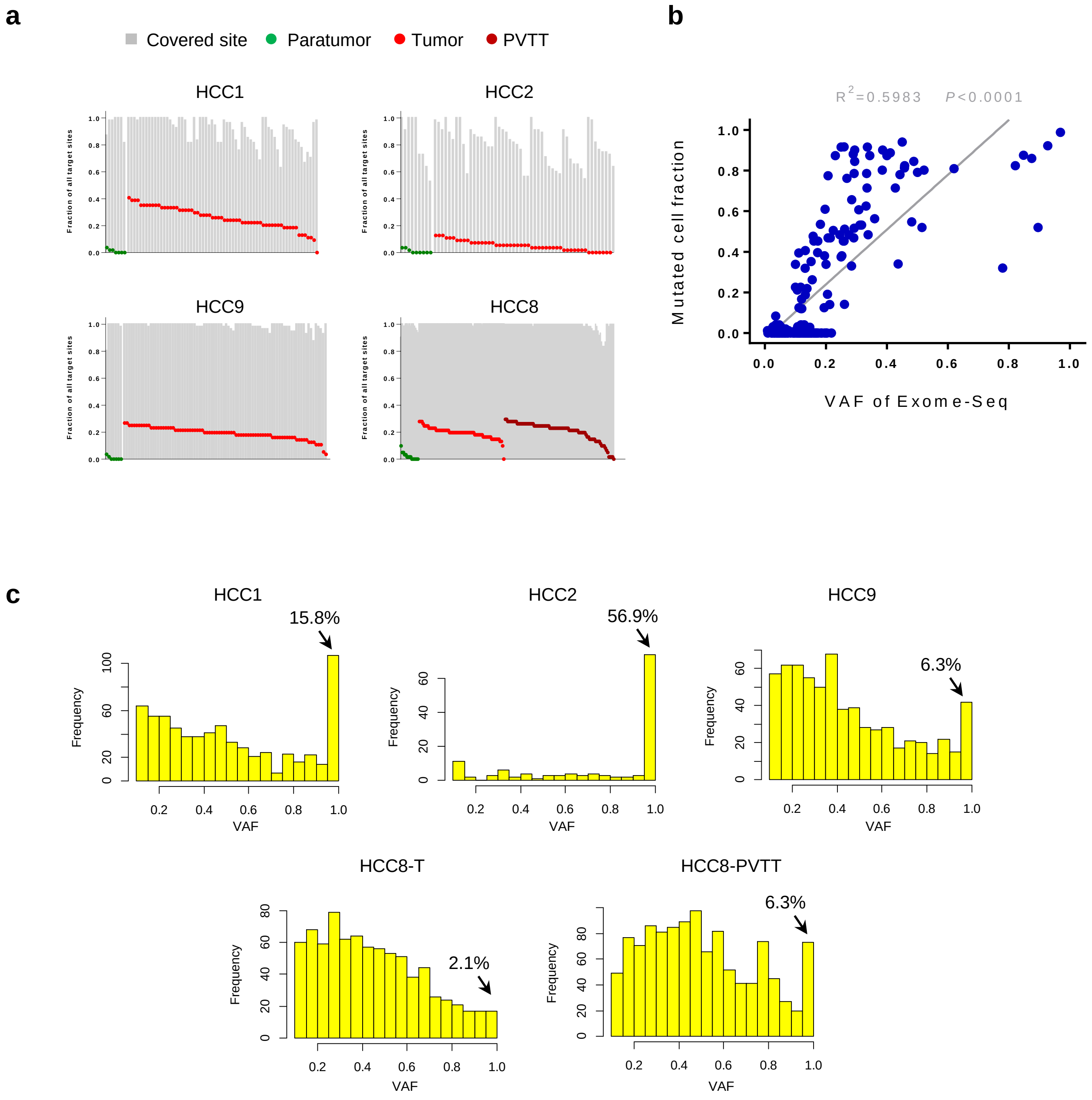

Supplementary Fig. 3 High quality single-cell mutation data were obtained by target sequencing. **a** Proportions of covered and mutated sites in each single cell in the 4 HCC patients. **b** Correlation plot between mutated cell fractions in single-cell target sequencing and WES-derived VAF values of mutations from HCC. Each dot represented a mutation, and the grey line represented linear regression with R^2 and *P* value shown. Please note that the copy number changes of target sites will cause deviations from standard correlation. **c** Histograms showing the distributions of VAF values in single cells among each HCC sample, after filtering those values below 0.1. The percentage of variations with VAF values between 0.95~1 were shown in each sample for allelic drop-out (ADO) assessment. Except for HCC2 with rate of 56.9%, all samples showed good results with low ADO rate.

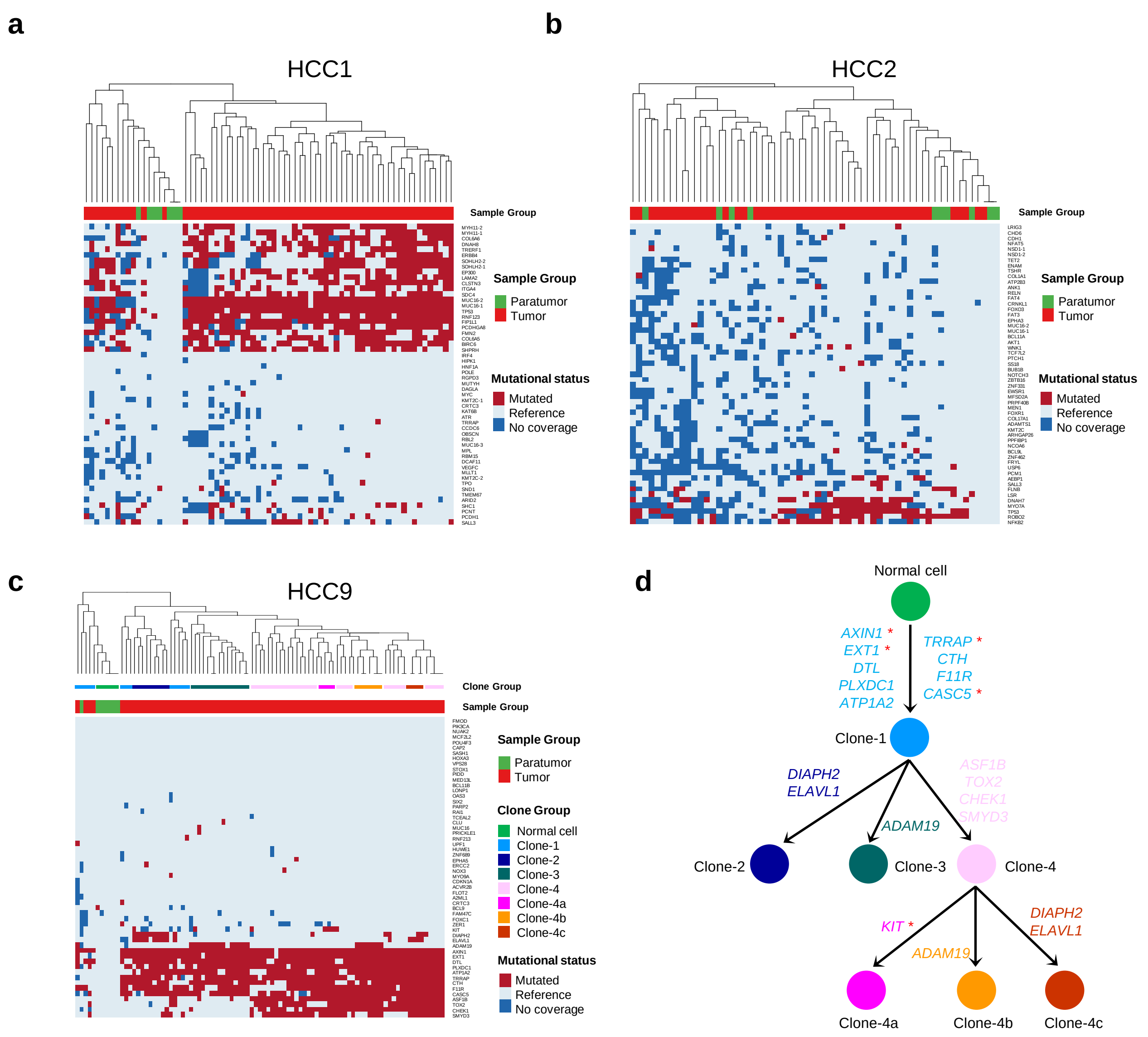

Supplementary Fig. 4 Single-cell clonal structures of HCC1, HCC2 and HCC9 based on point mutations. **a-c** Mutational status of SNV/INDEL sites in single cells from both paratumor and tumor tissues in HCC1 (**a**), HCC2 (**b**) and HCC9 (**c**). The inferred clone groups in HCC9 were also annotated. **d** Clonal evolution in HCC9 with genes mutated at each step shown. *: COSMIC Cancer Gene Census catalogued driver genes.

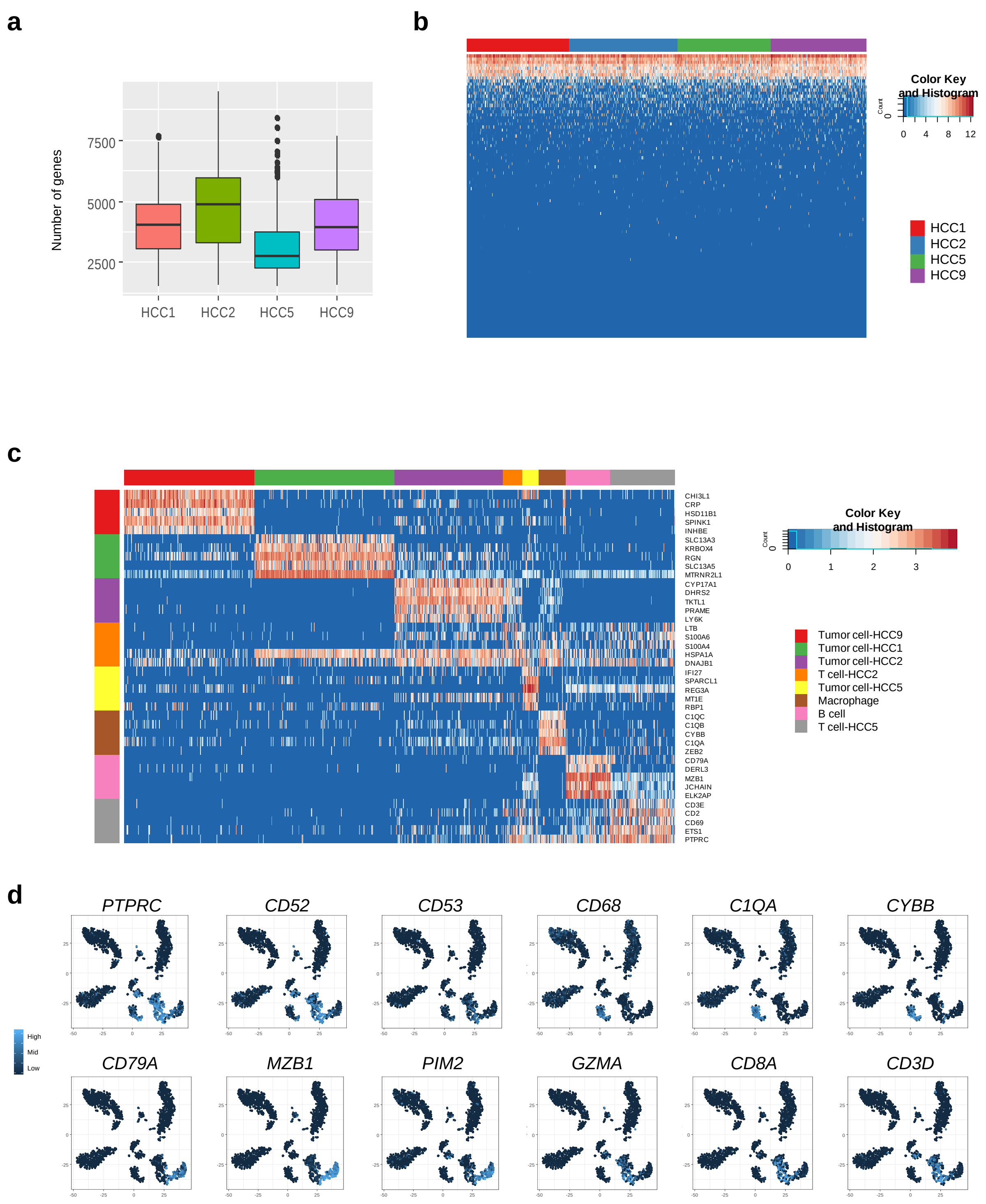

Supplementary Fig. 5 scRNA-Seq revealed the constituent cell types of HCC. **a** Distribution of gene numbers detected in scRNA-Seq of each patient, with boxes showing median and 1st and 3rd quartiles, and whiskers showing 1.5 times of the inter-quartile range. **b** Heatmap showing the expression of ERCC RNA Spike-ins. Each row represented a spike-in, and each column represented a single cell. **c** Heatmap showing the genes specifically expressed in each cell cluster. **d** The expression pattern of representative immune markers.

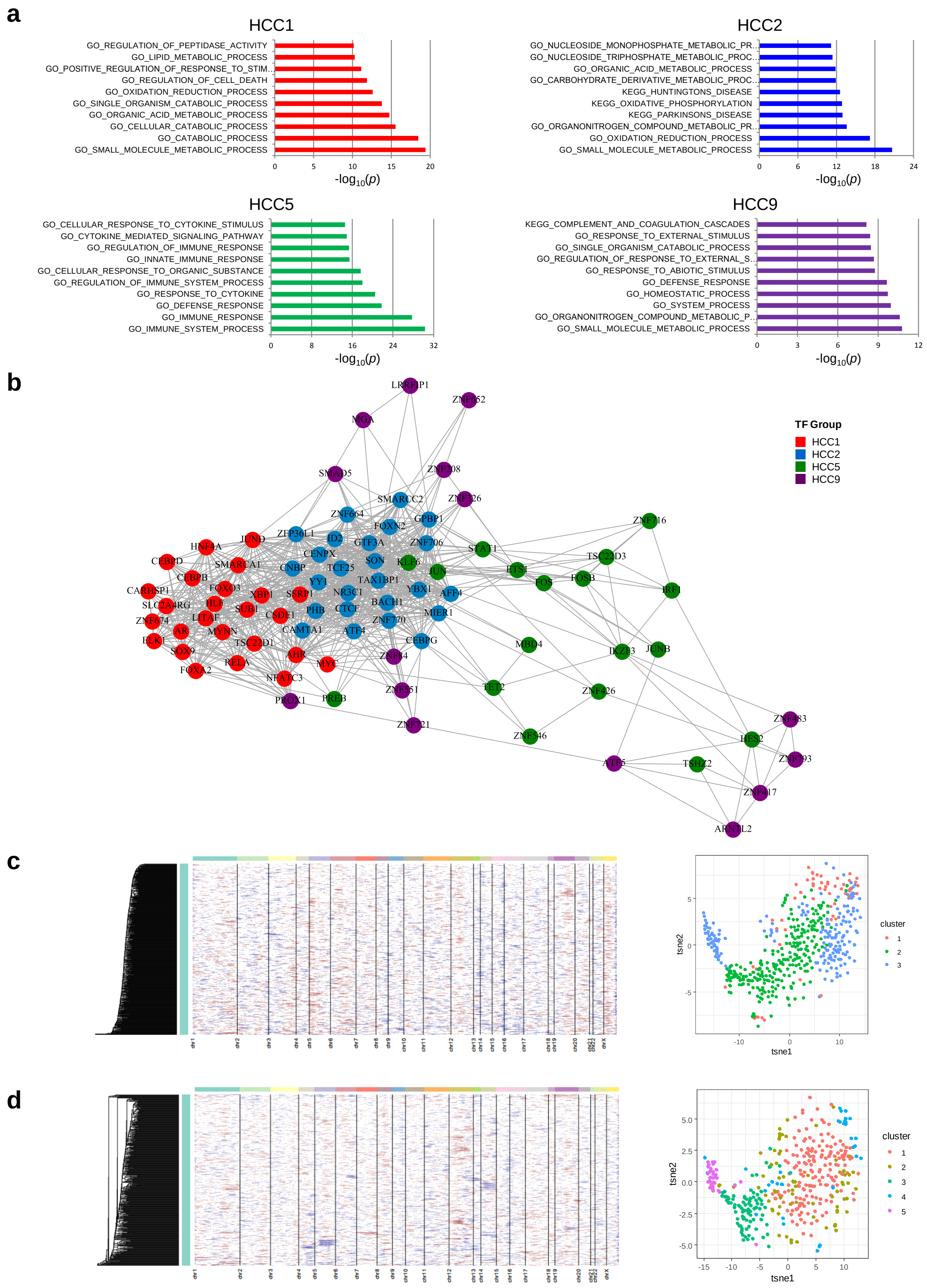

Supplementary Fig. 6 scRNA-Seq revealed the inter-tumor and intra-tumor heterogeneity of HCC. **a** The top 10 GO enrichment items for the genes specifically expressed in each tumor cluster. **b** Transcriptional factor covariance network of the HCC scRNA-Seq data. **c,d** The copy number changes inferred from global transcriptomic profiles of single cells (*left*) and the sub-populations based on differentially expressed genes (*right*) from HCC1 (**c**) and HCC2 (**d**).

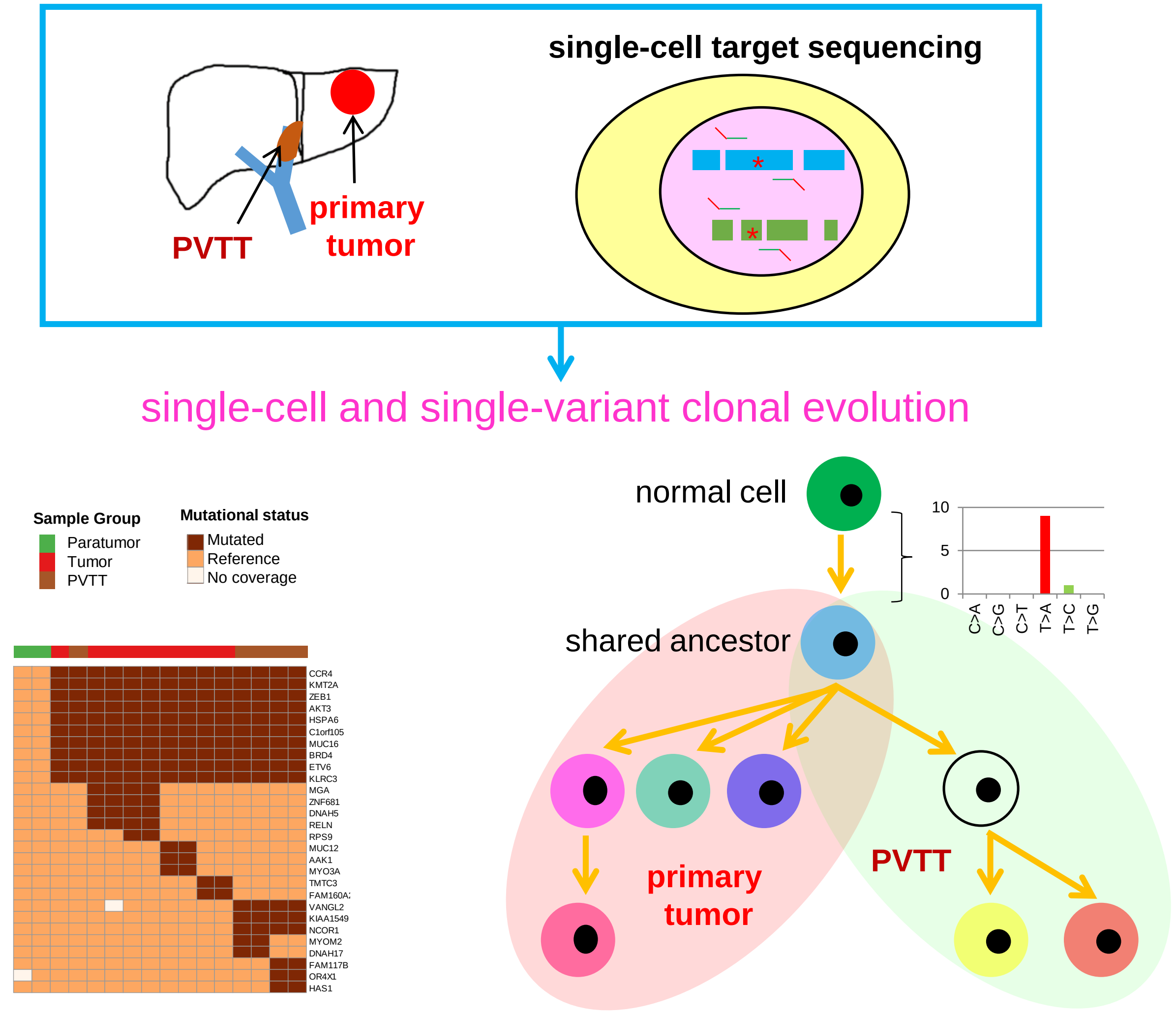

Supplementary Fig. 7 Schematic representation of major findings in this study.
